# Air Temperature and Relative Humidity Datasets from an Urban Meteorological Network in the City Area of Novi Sad (Serbia)

**DOI:** 10.1016/j.dib.2023.109425

**Published:** 2023-07-20

**Authors:** Stevan Savić, Ivan Šećerov, Branislava Lalić, Dongyun Nie, Mark Roantree

**Affiliations:** a Faculty of Sciences, University of Novi Sad, Trg Dositeja Obradovića 3, 21000 Novi Sad, Serbia; b Faculty of Agriculture, University of Novi Sad, Trg Dositeja Obradovića 8, 21000 Novi Sad, Serbia; c School of Computing, Insight Centre for Data Analytics, Dublin City University, Collins Ave, Dublin 9, Ireland

**Keywords:** Urban monitoring network, *In situ* measurement, Air temperature values, Relative humidity values, Intra/inter-urban relations, Spatio-temporal data

## Abstract

This data article describes two groups of datasets which capture, firstly - 10-minutes air temperature (T_a_) and relative humidity (RH) data from 27 urban and non-urban sites over a period of 3.5 years covering 2014–2018; and secondly - hourly T_a_ data from 12 urban sites over a period of 2 years covering 2016 and 2017. Both datasets are from urban meteorological network located in the Novi Sad city (Serbia). These datasets have 2 different types of information in the collection: one type provides details about the monitoring sites at which the T_a_ and RH sensors are placed, while the second type contains T_a_ and RH data at all sensor locations. In all, the 10-minutes dataset contains about 185,000 instances of T_a_ and RH data, and the hourly datasets contain 17,544 instances of T_a_ data. The 10-minutes datasets were not quality controlled, but the hourly T_a_ data has been cleaned and gap-filled so there are 24 measures at each site for each day. There are multiple potential uses, where this data can be applied. It can provide insights in understanding intra-urban and inter-urban research, urban climate modeling on local or micro scales, heat-related public health investigations and urban environment inquiries. It can also be used in machine learning experiments, for example, to test the accuracy of classification algorithms or to build and validate spatio-temporal machine learning functions, either for classification purposes or for gap filling. These datasets are directly citable through its DOIs and available for download from the Zenodo platform or from the Fair Micromet Portal.

**Specifications Table**

**Table:** 

Subject	Environmental Science: Climatology
Specific subject area	Urban climatology: *In situ* measured 10-minutes and hourly air temperature (T_a_) and relative humidity (RH) variables for intra- and inter-urban assessments and spatio-temporal analyses
Type of data	Comma separated text files (.csv) – T_a_ and RH datasets; Excel files (.xlsx) – metadata of station sites
How the data were acquired	Raw T_a_ and RH data were obtained from the Novi Sad Urban Network (NSUNET) wireless system located in the urban area of Novi Sad and its surrounding [1]. Data was collected from measurements in situ, using 27 measurement sites, each equipped with ChipCap 2 air temperature and relative humidity sensors developed by the General Electric Measurement & Control Company. Sensors were placed in ventilated radiation protection screens [1]. Datasets consisting of 10-minutes and hourly based T_a_ and RH values, obtained from 25 urban sites and 2 non-urban sites. 10-minutes datasets are raw and not quality controlled, but hourly T_a_ datasets from 12 urban stations are subjected to quality control procedures (detection of outliers and gap filling) [2].
Data format	Raw (Not quality controlled – with outliers and gaps) – 10-minutes T_a_ and RH datasets from 27 urban and non-urban sites. Raw (Quality controlled – no outliers and no gaps) – hourly T_a_ datasets from 12 urban sites.
Description of data collection	The outdoor T_a_ (in °C) and RH (in%) data were collected at 10-minutes basis for the period between 1st June 2014 and 8th February 2018. Data from sites in urban areas (25 stations) collected T_a_ and RH values from different built-up local climate zones (LCZs) and data from sites in non-urban areas collected T_a_ and RH values from different land cover LCZs [1]. The outdoor T_a_ data (in °C) was collected on an hourly basis for the period between 1st January 2016 and 31st December 2017. Data from sites in urban areas contains T_a_ values from different built-up LCZs [1].
Data source location	Institution: University of Novi Sad, Faculty of Sciences Region: Europe Country: Serbia City: Novi Sad Latitude and longitude for collected samples/data: 45°15′18″N, 19°50′41″E Domain size (km^2^): 102
Data accessibility	Repository name: Zenodo and Fair Micromet Portal (FMP) Data identification number for the 10-minutes T_a_ and RH datasets: doi:10.5281/zenodo.8114905 Direct URL to 10-minutes T_a_ and RH data: https://www.zenodo.org/record/8114905 Data identification number for the hourly T_a_ datasets: doi:10.5281/zenodo.7738094 Direct URL to hourly T_a_ data: https://doi.org/10.5281/zenodo.7738094 or https://fairmicromet.eu/networks/894abd82-c49c-4b5e-8506-f600362cb4da Instructions for accessing these data: The datasets are freely available for scientific and public purposes through the Zenodo and FMP platforms.
Related research article	I. Šećerov, S. Savić, D. Milošević, D. Arsenović, D. Dolinaj, S. Popov, 2019. Progressing urban climate research using a high-density monitoring network system. Environ. Monit. Assess. 191, 89. https://doi.org/10.1007/s10661–019–7210–0

## Value of the Data

• The T_a_ and RH data, obtained from the high-densely urban meteorological network, contributes to comprehensive urban thermal and climate analyses on seasonal or daily levels, and can be useful in detailed intra-urban and inter-urban research, urban climate modeling on local or micro scales, heat-related public health investigations and urban environment inquiries.
• Data provided by this research can be used for evaluations of fine-scale temperature and humidity models in urban areas, as well as material for risk studies of extreme weather events (heat or cold waves). It can provide insightful information used in local or regional climate change adaptation strategies in cities, benefitting climate researchers, data scientists, health policy professionals and urban planners.
• Researchers can use this data as it is well suited to both machine learning and time series researchers as data is of a spatio-temporal nature, suited to gap filling or classification machine learning models.
• Educators can use this data for machine learning (clustering, classification, time series analyses) in under-graduate and post-graduate training.
• Researchers in the field of climatology, meteorology and public health who are focused on the interactive effects of climate change and urbanization on population and environment in cities.
• The data can also be useful for stakeholders, especially urban planners, architects, demographers, environmentalists who investigate heat load effects on various social/urbanization activities in cities.

## 1 Objective

The datasets are obtained from the Novi Sad Urban Network (NSUNET) system that was created as a part of the international cross-border project [3], where each measuring site was equipped with multiple sensors and a variety of electronic and hardware devices. In creating this urban monitoring network, the project's objective was to provide conditions for progressive urban climate research into the future, i.e. contribute to the thermal pattern differences with an in-depth investigation of the various urban designs and city surroundings [1,^4^,5]. Ultimately, the primary motivation for creating the NSUNET network was to obtain conditions for further urban climate research by way of intra- and inter-urban research and thus, widening possibilities for cooperating with research groups having similar goals.

## 2 Data Description

### 2.1 Description of monitoring area

Novi Sad is the second largest city in the Republic of Serbia, with 102 km^2^ of built-up and urban green/blue areas and a population of 330,000 people in 2017. The city is located on the Pannonian Plain in Central Europe (45°15′18″N, 19°50′41″E), and thus, most of the urban area is flat with an absolute elevation between 72 m and 80 m [6]. Novi Sad has a *Cfb* climate (temperate climate, fully humid, warm summers, with at least four months of average *T_a_* above 10 °C) based on the Köppen-Geiger climate classification system [7].

### 2.2 Air temperature and relative humidity data

The NSUNET system generated two separate databases with different measurement frequencies (10-minutes and hourly) and different quality control protocols.

10-minutes T_a_ ( °C) and RH (%) data are obtained from the NSUNET system that covered urban and non-urban area of Novi Sad and its surroundings. These meteorological parameters are obtained from 27 stations with data from all stations presented in a single .csv file [1,8]. The content of the .csv file is organized in such a way that the first column represents *date* (dd/mm/yy), the second *time* (hour:minute), while the remaining columns represent *T_a_ and RH values* from each of 27 stations, respectively (e.g. 15.6 °C and 47.8 RH). The name (ID) of each station is defined by two digits: the first represents the number of the local climate zone (LCZ), and the second one represents the number of each sensor in a particular LCZ [1,9]. Each station provides 10-minutes measurements covering the time period from 1st July 2014 to 8th February 2018 using Coordinated Universal Time (UTC). Datasets are freely available on the Zenodo platform [8].

Hourly T_a_ data ( °C) are obtained from the NSUNET system that covered the urban area of Novi Sad. Hourly T_a_ datasets from NSUNET are located across 12 different urbanized sites and contain 12 temperature sensors (datasets from all sensors are presented in one .csv file) [1,10]]. The content of the .csv file is organized so that the first column represents *date* (dd/mm/yy), the second one *time* (hour:minute), while the remaining 12 columns represent *temperature values* from each sensor, respectively (e.g. 15.6). The name (ID) of each sensor is defined by two digits: the first represents the number of the LCZ, and the second one represents the number of each sensor in particular LCZ [1,9]. Each sensor provides hourly measurements covering the time period from 1st January 2016 to 31st December 2017 using the UTC. Datasets are freely available on Zenodo and FMP platforms [10,11], with WMO metadata descriptions on the Knowledge Sharing Platform constructed as part of a FAIRNESS Cost Action with FAIR testing protocols as described in [11,12].

In the following sections, the quantity and quality details for the hourly T_a_ datasets is presented. Note that for the 10-minutes interval of T_a_ and RH datasets, the same analysis on the presence of outliers and missing values, is not provided.

### 2.3 Metadata of 12 measurement sites and air temperature sensors

Table 1 displays site metadata of 12 T_a_ sensors (with hourly data) using 8 columns to describe the site locations for temperature sensors. The station ID corresponds to the station ID column in T_a_ metadata, with address, longitude and latitude. LCZ values range from 2 to 8 while the description is taken from 5 possible values. Station height refers to how high the sensor is located above ground while altitude refers to the specific site location.

**Table 1:** Site Metadata.

Variable Name	Type	Variable Description
Type	Categorical	{Urban, Rural}
Station_ID	Categorical	n-m, where n, m are integers and n has the same value as LCZ
Station_Address	Categorical	Text field
LCZ	Categorical	Integer value in range: 2–8
LCZ_Description	Categorical	{compact midrise, compact lowrise, open midrise, open lowrise, large lowrise,….}
Latitude	Numeric	Decimal (10,8)
Longitude	Numeric	Decimal (11,8)
Station_Height	Numeric	Decimal (3,2) (metres)
Altitude	Integer	Range: 75–92 (metres)

T_a_ metadata is shown in Table 2. There are 14 columns in all: date, time, and the remaining 12 columns contain the T_a_ for each site.

**Table 2:** Air temperature (T_a_) Metadata.

Variable Name	Type	Variable Description
Date	Date	1.1.2016–31.12.2017
Time	Time	00:00 to 23:00
s2–2	Integer	Range: −12, 40.2
s2–3	Integer	Range: −13.5, 41
s3–2	Integer	Range: −13.3, 40.2
s5–2	Integer	Range: −14.0, 40.2
s5–3	Integer	Range: −13.0, 40.1
s5–4	Integer	Range: −13.1, 39.8
s5–5	Integer	Range: −12.5, 39.2
s5–6	Integer	Range: −12.8, 40.7
s6–4	Integer	Range: −13.2, 40.1
s6–8	Integer	Range: −14.2, 40.0
s6–9	Integer	Range: −14.2, 40.9
s8–1	Integer	Range: −14.1, 41.9

### 2.4 Detailed summary description

For each sensor, 17,544 instances of hourly T_a_ have been recorded, with Table 3 showing a statistical description for hourly T_a_ *values* on a site basis. The top row represents the station names while rows provide values for: *Mean, standard deviation (std),* min, max*, 25%, 50% and 75% quartiles.*

**Table 3:** Statistical analysis for air temperature (T_a_) provided by 12 urban sites.

Station	s2–2	s2–3	s3–2	s5–2	s5–3	s5–4	s5–5	s5–6	s6–4	s6–8	s6–9	s8–1
Mean	14.3	13.7	13.7	13.9	13.8	13.5	14.2	14.4	13.6	13.3	13.4	14.2
STD	9.4	9.7	9.8	9.9	9.6	9.6	9.6	9.7	9.7	9.7	9.8	9.9
Min	−12.7	−13.5	−13.3	−14.0	−13.0	−13.1	−12.5	−12.8	−13.2	−14.2	−14.2	−14.1
25%	7.6	6.8	6.8	6.9	7.0	6.7	7.4	7.6	6.8	6.4	6.4	7.2
50%	14.2	13.4	13.6	13.7	13.6	13.4	14.0	14.3	13.5	13.1	13.1	14.1
75%	21.5	20.8	20.8	21.2	20.8	20.6	21.4	21.5	20.5	20.4	20.3	21.3
Max	40.2	41.0	40.2	40.2	40.1	39.8	39.2	40.7	40.1	40.0	40.9	41.9

**Fig 1:**
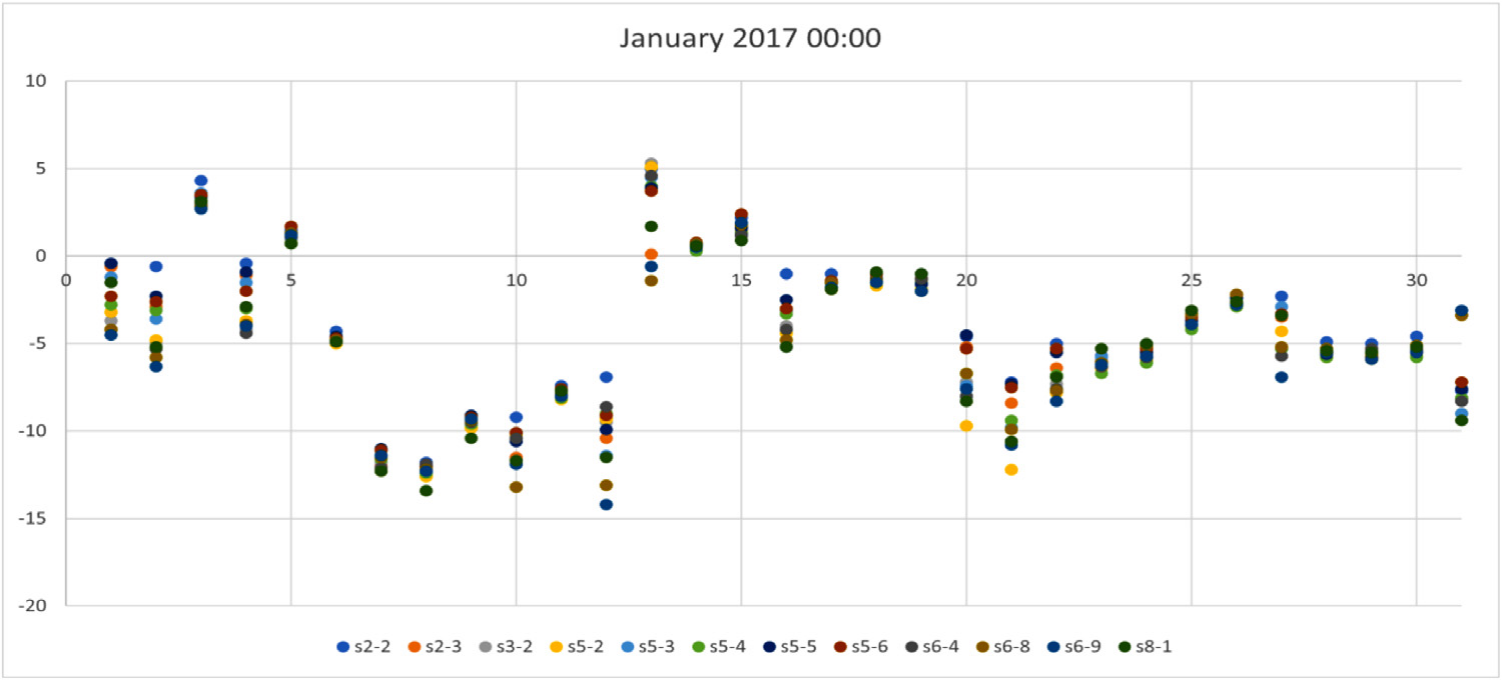
Temperature at midnight in January 2017 across the 12 urban sites that obtained the hourly T_a_.

**Fig 2:**
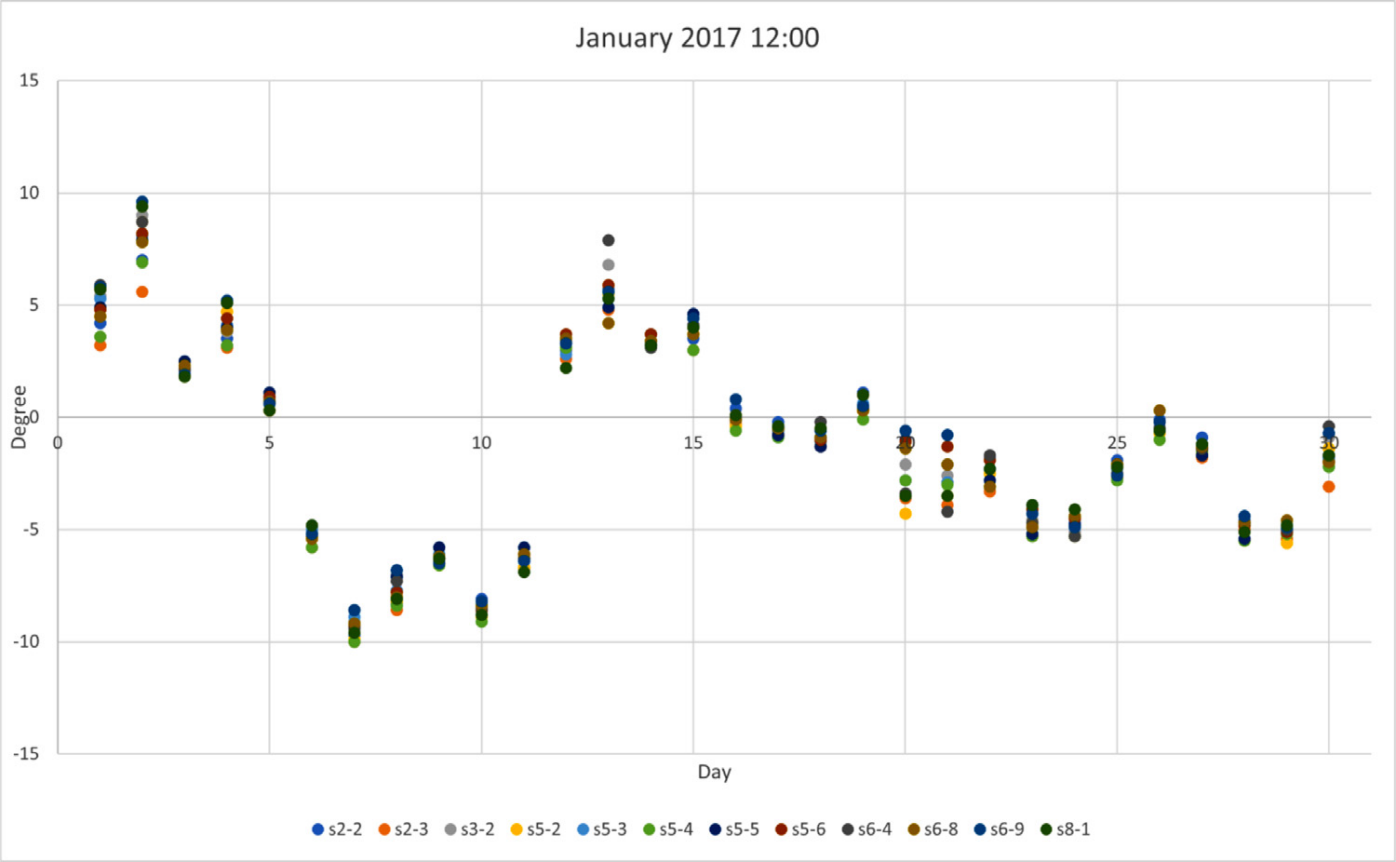
Temperature at midday in January 2017 across the 12 urban sites that obtained the hourly T_a_.

**Fig 3:**
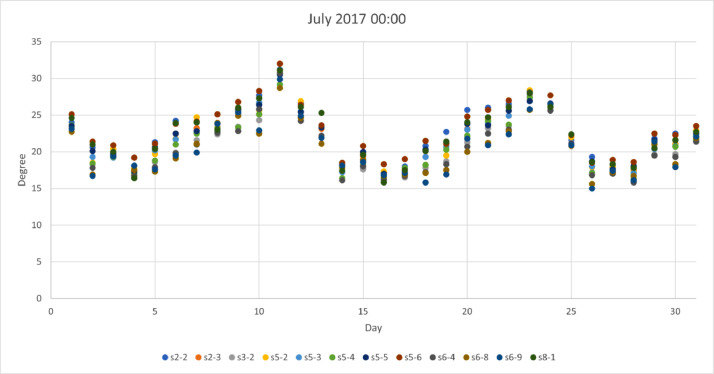
Temperature at midnight in July 2017 across the 12 urban sites that obtained the hourly T_a_.

**Fig 4:**
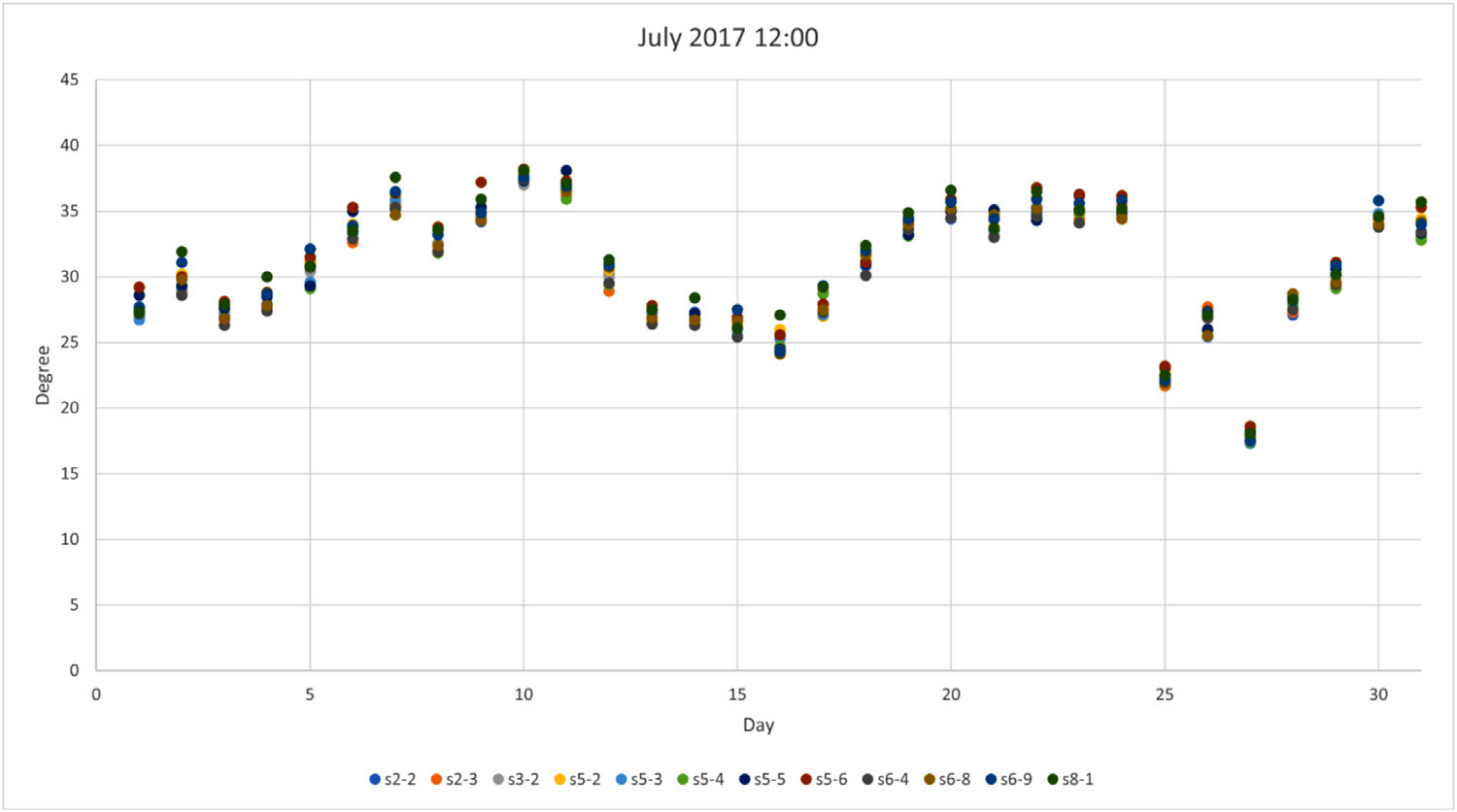
Temperature at midday in July 2017 across the 12 urban sites that obtained the hourly T_a_.

We provide a visual sample from Table 3 in Fig 1, Fig 2, Fig 3, Fig 4, where winter (January) and summer (July) hourly T_a_ for both midnight and midday are shown for 2017. In these figures, the *x*-axis represents the day (31 values in each case) for the selected month and the *y*-axis represents the T_a_. Each site has a selected color which can be used to identify those sites that are warmer or colder than others. These four figures are also useful to visualize the range of T_a_ across sites for a specific date in winter or summer.

## 3 Experimental Design, Materials and Methods

### 3.1 Raw data collection

**Fig 5:**
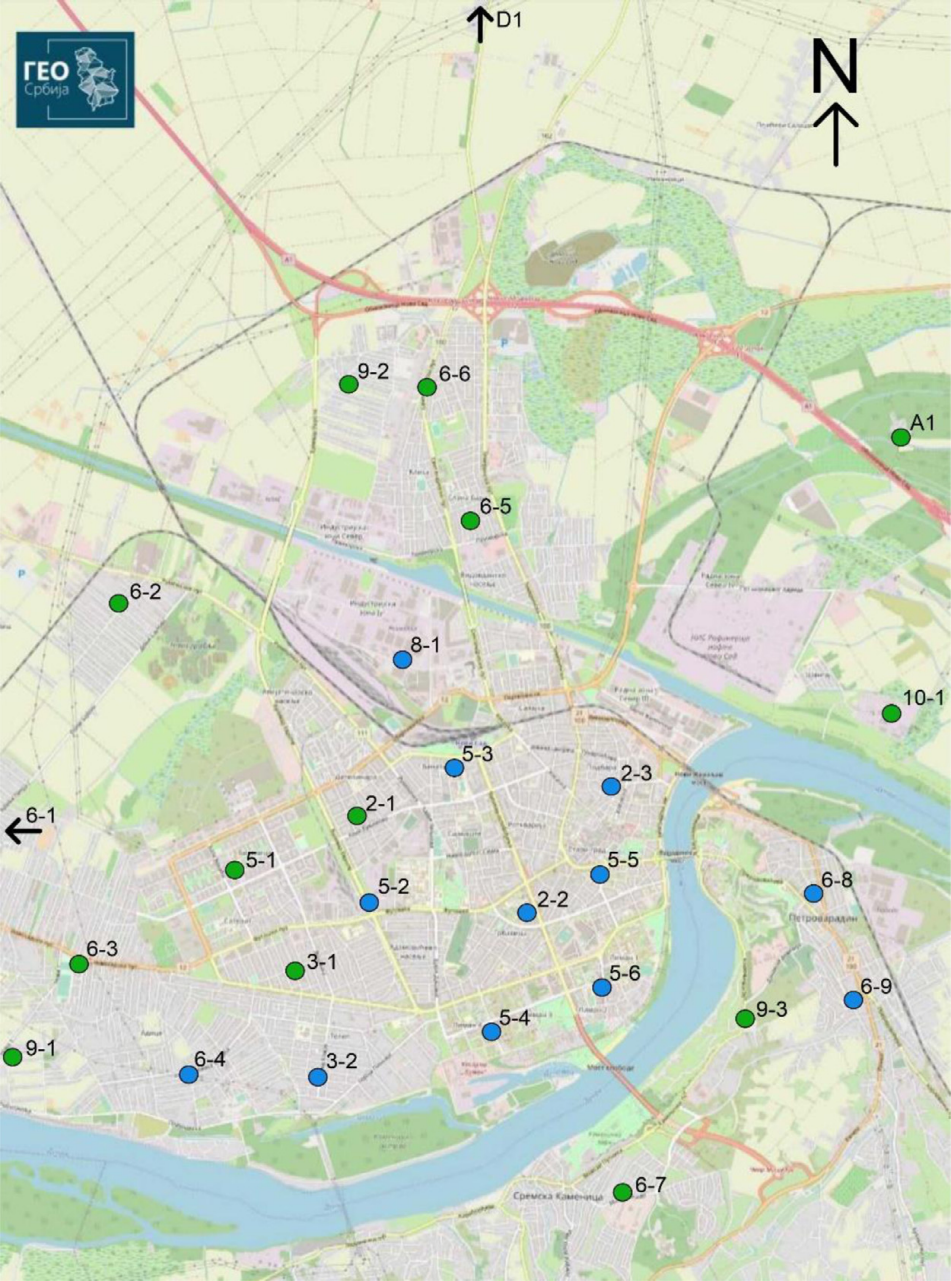
NSUNET station sites in urban Novi Sad. Blue dots – hourly T_a_ data from 12 urban sites; Blue + green dots – 10-minutes T_a_ and RH data from 27 urban and non-urban sites. Map Source: https://a3.geosrbija.rs/.

Sites for each sensor were selected to represent a thermal/humidity pattern across different built-up areas and their surroundings, as shown in Fig. 5. These urban area types are known as the LCZs, and were proposed by Stewart and Oke [9]. Using this method, the network was developed by selecting sensor locations based on two main criteria: firstly, that stations be evenly distributed based on the ratio of each LCZ within urban area, while at the same time, each station should be 100–200 m inside the defined LCZ; secondly, that the station be positioned in a street location to maximize protection from vandalism. The LCZ map with T_a_ and RH sensor sites were presented in the study of Šećerov et al. [1].

NSUNET continuously collected raw T_a_ and RH values from July 2014 to February 2018. Data was collected using ChipCap 2 sensors, fully calibrated and developed by the General Electric Measurement & Control Company, and located in a ventilated radiation protection screen with dimensions of 200 × 240 mm. Based on the calibration certificate provided by the manufacturer, further calibration of sensors was deemed unnecessary during the period of network operation. The accuracy of the T_a_ sensor was ±0.3 °C and the RH sensor was ±2% (20–80% RH), and they were installed at least 4 m above ground (with exceptions ±0.2 m), on arms (50 cm long) fixed to selected lamp posts. Each measurement site, near the sensors, was equipped with a station containing a central processor, EPROM chip used for data storage, GPRS/EDGE/3 G modem, backup battery and charger. Sensors at city sites had a direct power supply with batteries charged during the time that street lights were powered on. Sensors measured the T_a_ and RH values every minute *and* every 10 min, with measured data sent to the main server located at University of Novi Sad, Faculty of Sciences. At each site, the internal memory for the cloud storage approximated 700,000 measurements (15 months), meaning more consistent datasets in the face of problems due to mobile Internet provider or server issues [1,13]. The network ceased to operate in February 2018 due to the lack of institutional financial support for operating the network (particularly the cost of data transfer) and lack of support from government institutions to maintain the hardware.

**Table 4:** Distance (in km) between sensors based on 12 urban temperature sensors provided hourly T_a_ values.

	s2–2	s2–3	s3–2	s5–2	s5–3	s5–4	s5–5	s5–6	s6–4	s6–8	s6–9	s8–1
s2–2	0	1.64	2.78	1.66	1.71	1.28	0.91	1.08	3.94	3.01	3.57	2.88
s2–3	1.64	0	4.37	2.86	1.77	2.88	0.93	2.10	5.42	2.37	3.42	2.51
s3–2	2.78	4.37	0	1.92	3.50	1.88	3.68	3.11	1.39	5.53	5.65	4.43
s5–2	1.66	2.86	1.92	0	1.61	1.86	2.48	2.57	2.63	4.66	5.20	2.51
s5–3	1.71	1.77	3.50	1.61	0	2.76	1.96	2.76	4.19	4.04	4.93	1.18
s5–4	1.28	2.88	1.88	1.86	2.76	0	2.03	1.23	3.24	3.66	3.79	3.93
s5–5	0.91	0.93	3.68	2.48	1.96	2.03	0	1.17	4.86	2.20	2.98	2.99
s5–6	1.08	2.10	3.11	2.57	2.76	1.23	1.17	0	4.44	2.43	2.66	3.91
s6–4	3.94	5.42	1.39	2.63	4.19	3.24	4.86	4.44	0	6.84	7.02	4.87
s6–8	3.01	2.37	5.53	4.66	4.04	3.66	2.20	2.43	6.84	0	1.28	4.88
s6–9	3.57	3.42	5.65	5.20	4.93	3.79	2.98	2.66	7.02	1.28	0	5.90
s8–1	2.88	2.51	4.43	2.51	1.18	3.93	2.99	3.91	4.87	4.88	5.90	0

The distance (kms) between 12 urban sensors is presented in Table 4 as a dissimilarity matrix, computed using the Haversine function. The Haversine distance is the distance between two points on the surface of a sphere where the coordinates of each point are the (latitude, longitude) pair. The result in radians is then converted to kms. This type of matrix is used by a number of clustering algorithms to determine how close 2 points are to each other. This is useful, for example, when using spatial data to fill gaps as one can set a distance threshold beyond which, sites are not reliable in providing gap filling support.

### 3.2 Technical specifications for network development

In terms of implementation, NSUNET used the file transfer protocol (FTP) to send measured data to the servers using a predefined structure. FTP, a well-established protocol of choice, is highly reliable for file transfer. One of the main objectives was to develop a back-end system (defined as Core Segment) sustainable for an extended period and adaptable to any operating system changes. Variable names were defined with 2–3 capital letters, followed by character ‘:’ and its measured value. Each measurement is terminated using Unix EOL, character (0xa). Using this data structure provided a plaintext view of measured data while allowing a fast file parsing process. Two variable types were introduced in NSUNET's design: a) climatological and b) debug. The former stored urban-climate data while the latter produced information on the entire workings of the wireless network of stations (defined as Remote Segment). The file delivered to the system used the naming convention: station-id_date_time.txt. The Core Segment contained configuration files for each station, with the same data structure design. Thus, stations could be re-configured remotely. Each station was defined with its own unique ID while each measurement contained its measurement session (MS) together with time of measurements (time stamp, TS). There were roughly 28 different variables used in the ASCII (plaintext) data structure. Data were subjected to different QC methods as part of the core segment modules. The Core Segment itself was developed using open-source technologies, more specifically BASH shell scripting. To ensure the highest levels of reliability of each point of failure (from sensor up to the database server instance), an additional study was performed [13]. A highly reliable server-client model was developed to support this form of experimental study. Here, the Core Segment was stress tested with roughly 20,000 parallel connections. Above this, the reliability of station data degrades with frequent and different errors in inter-process communications (IPC) of the Linux systems.

### 3.3 Outlier analysis and gap filling

From the T_a_ raw values, measured at a 10-minute frequency, 1-hour datasets were extracted for the period 1st January 2016 - 31st December 2017, and subjected to quality control (QC) measures. The QC methodology comprised two main steps: logical outliers were removed and missing temperature values were interpolated. The outlier detection process excluded all values higher than 50 °C or lower than −30 °C. Gap filling comprised three sub-methods based on linear interpolation. The complete description of the quality control methodology i.e. outlier detection and gap filling, are explained in the guideline [2].

## Ethics Statements

The work did not involve the use of human subjects, animal experiments and data collected from social media platforms.

## CRediT Author Statement

**Stevan Savić:** Conceptualization, Writing – original draft preparation, Visualization; **Ivan Šećerov:** Visualization, Database creation, Investigation; **Branislava Lalić:** Writing – review & editing, Supervision; **Dongyun Nie:** Formal Analysis, Visualization; **Mark Roantreee:** Conceptualization, Writing – review & editing, Validation.

## Data Availability

Hourly Air Temperature Datasets from city of Novi Sad - NSUNET system (Original data) (Zenodo). 10-minutes Air Temperature and Relative Humidity Datasets from city of Novi Sad - NSUNET system (Original data) (Zenodo).
